# Epilepsy Surgery Series: A Study of 502 Consecutive Patients from a Developing Country

**DOI:** 10.1155/2014/286801

**Published:** 2014-01-30

**Authors:** Abdulaziz Alsemari, Faisal Al-Otaibi, Salah Baz, Ibrahim Althubaiti, Hisham Aldhalaan, David MacDonald, Tareq Abalkhail, Miguel E. Fiol, Suad Alyamani, Aziza Chedrawi, Frank Leblanc, Andrew Parrent, Donald Maclean, John Girvin

**Affiliations:** ^1^Department of Neurosciences, King Faisal Specialist Hospital and Research Centre, Riyadh 11211, Saudi Arabia; ^2^Neurology Section, Department of Neurosciences, King Faisal Specialist Hospital & Research Centre, MBC.76, P.O. Box 3354, Riyadh 11211, Saudi Arabia; ^3^University of Minnesota Medical Center, Fairview, Epilepsy Care Center, Minnesota, MN 55455, USA; ^4^University of Calgary, AB, Canada T2N 1N4; ^5^London Health Science Center, London, ON, Canada N6G 2V4

## Abstract

*Purpose*. To review the postoperative seizure outcomes of patients that underwent surgery for epilepsy at King Faisal Specialist Hospital & Research Centre (KFSHRC). *Methods*. A descriptive retrospective study for 502 patients operated on for medically intractable epilepsy between 1998 and 2012. The surgical outcome was measured using the ILAE criteria. *Results*. The epilepsy surgery outcome for temporal lobe epilepsy surgery (ILAE classes 1, 2, and 3) at 12, 36, and 60 months is 79.6%, 74.2%, and 67%, respectively. The favorable 12- and 36-month outcomes for frontal lobe epilepsy surgery are 62% and 52%, respectively. For both parietal and occipital epilepsy lobe surgeries the 12- and 36-month outcomes are 67%. For multilobar epilepsy surgery, the 12- and 36-month outcomes are 65% and 50%, respectively. The 12- and 36-month outcomes for functional hemispherectomy epilepsy surgery are 64.2% and 63%, respectively. According to histopathology diagnosis, mesiotemporal sclerosis (MTS) and benign CNS tumors had the best favorable outcome after surgery at 1 year (77.27% and 84.3%, resp.,) and 3 years (76% and 75%, resp.,). The least favorable seizure-free outcome after 3 years occurred in cases with dual pathology (66.6%). Thirty-four epilepsy patients with normal magnetic resonance imaging (MRI) brain scans were surgically treated. The first- and third-year epilepsy surgery outcome of 17 temporal lobe surgeries were (53%) and (47%) seizure-free, respectively. The first- and third-year epilepsy surgery outcomes of 15 extratemporal epilepsy surgeries were (47%) and (33%) seizure-free. *Conclusion*. The best outcomes are achieved with temporal epilepsy surgery, mesial temporal sclerosis, and benign CNS tumor. The worst outcomes are from multilobar surgery, dual pathology, and normal MRI.

## 1. Introduction

The incidence of epilepsy in developed countries is currently estimated to be 57 people per 100,000 inhabitants [1], and the prevalence of active epilepsy is between 3 and 8 people per 1,000 citizens [2–4]. The rate for active epilepsy in Saudi Arabia is 6.54 per 1,000 [5]. In Saudi Arabia, epilepsy disorders are common, with a hospital frequency rate of 8 per 1,000. Men were more frequently affected than women, and 60% of the patients were under 10 years of age at the onset of the illness [6].

Seventy to 80% of epilepsy patients can be satisfactorily managed with anticonvulsive medication [7] while 20% to 30% develop medically intractable epilepsy [8]. Epilepsy surgery started in the 19th century, but significant contributions were made to the procedure in the 20th century [9]. Currently, epilepsy surgery is the standard treatment for patients with refractory epilepsy. The effectiveness of surgery over constant pharmacotherapy for intractable epilepsy has been established in a randomized controlled trial [10]. Results from a controlled trial justified the use of surgery as soon as 2 years after the development of pharmacoresistance [11, 12]. Published evidence showed a significantly better quality of life for the patient, which certainly balances the somewhat minor risks associated with epilepsy surgery [13, 14].

Epilepsy surgery procedures were initiated in Saudi Arabia in 1998. Our first evaluation of these procedures was published in *Epilepsy*: a comprehensive textbook (chapter 296). This study, however, was an early systematic review of epilepsy surgery outcomes and related variables in the comprehensive epilepsy program at a single center (KFSH&RC) in Saudi Arabia. The focus of this current study is to review the postoperative seizure outcomes of patients that underwent epilepsy surgery at our centre over the 14-year period from 1998 to 2012. The clinical semiology, preoperative investigations, histopathological diagnoses, and surgical procedures were identified in the determination of outcomes.

## 2. Methods 

### 2.1. Study Design

A descriptive retrospective study was conducted using data from the epilepsy registry database of our epilepsy program. The study analyzed all patients operated on between 1998 and 2012. The inclusion criteria for surgical evaluation were as follows: (1) recurrent partial seizures with or without secondary generalized seizures and (2) failure of at least two first-line antiepileptic drugs to control the seizures [15].

### 2.2. Presurgical Evaluation

The preoperative evaluation includes a neurological examination, MRI video-EEG recording with electrodes placed according to the International 10–20 system, invasive video-EEG recording, a neuropsychological evaluation, a sodium amobarbital test, and nuclear brain-scan studies. Once the evaluation is completed, the data are usually discussed in a multidisciplinary patient management conference, which includes epileptologists, neurosurgeons, a neuroradiologist, and a neuropsychologist. A standard epilepsy MRI protocol was used to study all patients (GE 1.5 and 3 T; GE Sigma Excite 1.5 T): sagittal T1 sequences (5 mm slice thickness), coronal FLAIR (5 mm), T2 (3 mm), axial T1, T2, and T2* (5 mm), 3D T1 sequences, and diffusion of 1.6 mm slice thickness. Video-EEG monitoring with intracranial electrode implants was also used prior to surgery. Interictal FDG-PET scan was used to evaluate surgical cases. Neuropsychological evaluation was done using a comprehensive test battery and following clinical diagnostic requirements. In contrast to the inclusion criteria for surgical evaluation (*vide supra*), the selection criteria for epilepsy surgery includes (1) a confirmed diagnosis of epilepsy, (2) the presence of medically intractable or disabling seizures, (3) a concordance of the localization data to a respectable focus, (4) the presence of a nonprogressive underlying disease (except Rasmussen's encephalitis), and (5) high probability that seizure control will significantly improve the patient's quality of life.

### 2.3. Surgical Procedures

The epilepsy procedures were classified as follows: (1) temporal lobe surgery (it includes the anterior temporal lobe with amygdalohippocampectomies and lesionectomies in anterior, middle temporal lobe, posterior lateral, and basal posterior temporal regions), (2) frontal lobe surgery, (3) parietal lobe surgery, (4) occipital lobe surgery, (5) functional hemispherectomy, (6) multilobar surgery (frontotemporal, temporoparietal, frontoparietal, temporoparietooccipital, parietooccipital, and temporooccipital), (7) anterior 2/3 callostomy, and (8) multiple subpial transaction.

### 2.4. Postsurgical and Outcome Assessment

Epilepsy surgery outcomes were measured according to the ILAE classification. Seizure-free patients with or without auras and patients with 1–3 seizures per year were considered to have favorable outcomes while more than 3 seizures per year were considered unfavorable (Table 1). Epilepsy surgery outcomes were assessed at 12, 36, and 60 months for temporal lobe surgery and at 12 and 36 months for the other procedures. The outcomes were measured using the parameters of procedure, histopathology (mesial temporal sclerosis, benign CNS tumor, cortical dysplasia, and dual pathology), and normal MRI brain.

### 2.5. Histopathology

The histopathology results were classified into one of the following nine categories: mesial temporal sclerosis (MTS), cerebral dysgenesis (focal cortical dysplasia and heterotopia), primary CNS tumor, vascular malformation, chronic inflammation (*Rasmussen encephalitis*), gliosis, dual pathology (i.e., the association of two potentially epileptogenic features), non specific and normal.

## 3. Results

### 3.1. Patients' Demography and Epilepsy Procedures

A total of 502 patients (296 male, 206 female) underwent surgery for their refractory epilepsy between 1998 and 2012. Out of those 502 patients, 65.3% were adults, and 34.7% were pediatric. The epilepsy surgical procedures included 295 temporal lobe surgeries, 53 frontal lobe surgeries, 16 parietal and occipital lobe surgeries, 64 functional hemispherectomies, 26 multilobar surgeries, 10 corpus callosotomies, and 3 multiple subpial transections and one hypothalamic hamartoma resection. The surgical outcome assessments of 468 patients that completed at least 3 years after surgery were evaluated. The invasive EEG was used in 141 patients (28%). The invasive EEG procedure was performed in patients with intractable epilepsy in whom the clinical analysis, scalp EEG, MRI, PET scan, and neuropsychology failed to lateralize seizure origin confidently.

### 3.2. Neuroimaging Results

MRI brain scans revealed 202 MTS cases, 78 primary CNS tumors, 66 cerebral cortical dysgenesis, 35 normal MRI, 66 focal atrophy and encephalomalacia, 22 arachnoid and *porencephalic* cysts, 15 vascular malformations, 11 tuberous scleroses, and 7 others. A PET scan was conducted on 398 patients, which showed 358 with hypometabolism, 21 with hypermetabolism, and 19 as normal.

### 3.3. Histopathology Results

The histopathology results showed 11 patients were normal, 203 had MTS, 142 had a cortical dysplasia/heterotopia, 96 had CNS tumors, 11 had encephalomulacia, 11 had chronic inflammations, 12 had vascular malformations, and other 16 (6 nonspecific and 10 cases of callosotomy). Thirty-eight of the histopathology results disclosed a dual pathology, each of which might have contributed to, or been responsible for, the epilepsy.

## 4. Epilepsy Surgery Outcomes according to Procedure

### 4.1. Temporal Lobe Surgery

**Figure 1:**
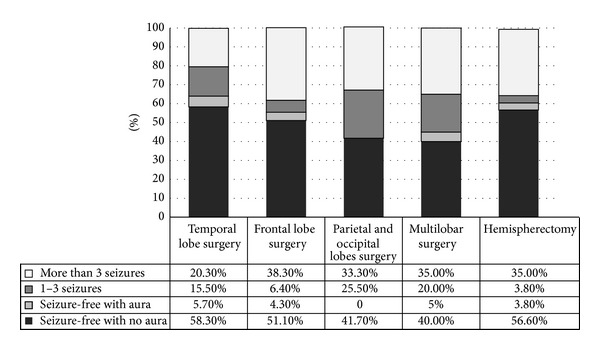
first-year epilepsy surgery outcome according to procedure.

**Figure 2:**
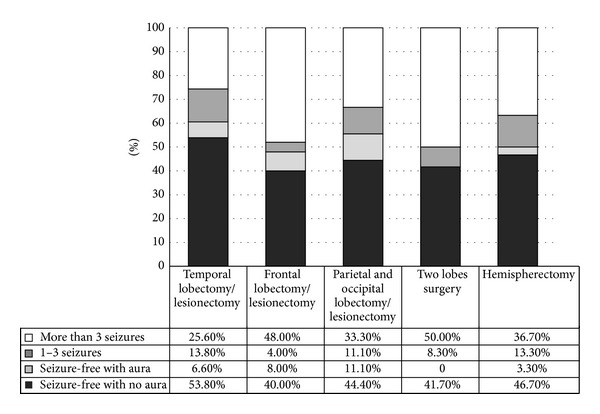
The third-year epilepsy surgery outcome according to procedure.

**Figure 3:**
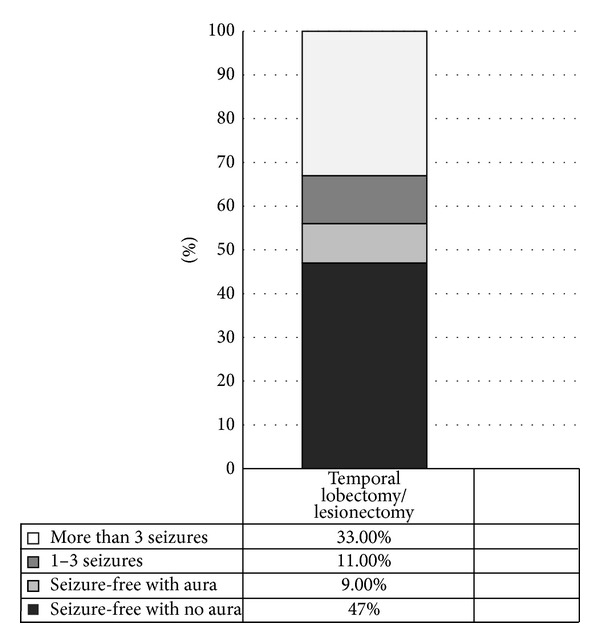
The fifth year epilepsy outcome temporal lobe surgery.

According to the ILAE, the favorable first-year epilepsy surgery outcome for patients following temporal lobe surgery is 79.6%; in our study, 172 patients (58.3%) were seizure-free without auras, 17 (5.7%) were seizure-free with auras, and 46 (15.5%) had 1–3 seizures per year. The favorable outcome for the third year following temporal lobe surgery, according to ILAE criteria, is 74.2%. At this point, our study found 105 patients (53.8%) were seizure-free without auras, 13 patients (6.6%) were seizure-free with auras, and 27 patients (13.8%) had 1–3 seizures per year. According to ILAE, the favorable outcome for the fifth year following temporal lobe surgery was 67%; in our series, 47 patients (47%) were seizure-free without auras, 9 (9%) were seizure-free with auras, and 11 (11.0%) had 1–3 seizures per year (Figures 1, 2, and 3).

### 4.2. Frontal Lobe Surgery

The favorable outcome for patients after the first year following epilepsy surgery is 62% according to ILAE criteria. Our study found 24 patients (51.1%) were seizure-free without auras, 2 patients (4.3%) were seizure-free with auras, and 3 patients (6.4%) suffered from 1–3 seizures per year. According to the ILAE, the favorable outcome for the third year following frontal lobe surgery is 52%; in our series, 10 patients (40.0%) were seizure-free without auras, 2 (8%) were seizure-free with auras, and 1 (4.0%) with 1–3 seizures per year (Figures 1 and 2).

### 4.3. Parietal and Occipital Lobe Surgery

According to the ILAE, the favorable first-year outcome for patients following parietal and occipital surgery is 67%. Our results found 5 patients (41.6%) were seizure-free without auras and 3 patients (25.5%) had 1–3 seizures per year. The favorable outcome in the third year following parietal and occipital surgery is 66.6%; our cases showed 4 patients (44.4%) were seizure-free without auras, 1 (11.1%) was seizure-free with auras, and 1 (11.1%) had 1–3 seizures per year (Figures 1 and 2).

### 4.4. Multilobar Surgery

The favorable outcome for patients in the first year following multilobar surgery, according to ILAE criteria, is 65%. Our study found 8 patients (40%) were seizure-free without auras, 1 (5%) was seizure-free with auras, and 4 (20%) had 1–3 seizures per year. According to ILAE, the favorable outcome for the third year following multilobar surgery, according to the ILAE, is 50%; in our cases, we had 5 patients (41.7%) that were seizure-free without auras, and 1 (8.3%) had 1–3 seizures per year (Figures 1 and 2).

### 4.5. Functional Hemispherectomy

According to ILAE criteria, the favorable outcome for patients in the first year following a hemispherectomy is 64.2%. Our results found 30 patients (56.6%) were seizure-free without auras, 2 (3.8%) were seizure-free with auras, and 2 (3.8%) had 1–3 seizures per year. The favorable outcome for the third year following a hemispherectomy, according to the ILAE, is 63%. Our study found 14 patients (46.7%) were seizure-free without auras, 1 (3.3%) was seizure-free with auras, and 4 (13.3%) had 1–3 seizures per year (Figures 1 and 2).

**Figure 4:**
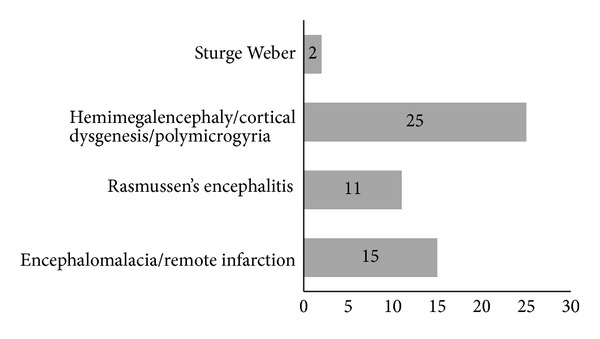
The underlying epilepsy disease of functional hemispherectomy cases.

A total of 53 functional hemispherectomies were performed in our series. The MRI brain diagnosis disclosed 11 cases of Rasmussen encephalitis, 25 of cerebral cortical dysgenesis and hemimegalencephaly, 15 of remote infarction and encephalomalacia (gliosis), and 2 of Sturge Weber disease. Forty-one of the patients were children (Figure 4).

## 5. Pediatric Epilepsy Surgery Outcome

146 children with refractory epilepsy underwent epilepsy surgery. The favorable first-year epilepsy surgery outcome for temporal lobe surgery according to ILAE criteria is 88.4%, (48 (69.6%) were seizure-free without aura, 2 (2.8%) seizure-free with aura, and 11 (16%) 1–3 seizures per year). The favorable third year for temporal lobe surgery (ILAE) is 72%; (25 (58%) seizure-free without aura, 4 (9%) seizure-free with aura, and 2 (5%) with 1–3 seizures per year). The favorable fifth year for temporal lobe surgery (ILAE) is 71%; (12 (57%) seizure-free without aura, 3 (14%) seizure-free with aura).

The favorable first year epilepsy surgery outcome for frontal lobe surgery according to ILAE criteria is 70% (11 (55%) were seizure-free without aura, 1 (5%) seizure-free with aura, and 2 (10%) 1–3 seizures per year). The favorable third year for frontal lobe surgery (ILAE) is 70% (6 (60%) seizure-free without aura and 1 (10%) seizure-free with aura).

The favorable first-year epilepsy surgery outcome for parietal and occipital lobes surgery according to ILAE criteria is 50% (2 (50%) were seizure-free without aura). Only one case completed three years after surgery and the outcome was seizure-free without aura.

The favorable first-year epilepsy surgery outcome for multilobar surgery according to ILAE criteria is 80%, (3 (60%) were seizure-free without aura and 1 (20%) with 1–3 seizures per year).

The favorable first-year epilepsy surgery outcome for functional hemispherectomy according to ILAE criteria is 60% (22 (55%) were seizure-free without aura, 1 (2.5%) seizure-free with aura, and 1 (2.5%) 1–3 seizures per year). The favorable third-year epilepsy surgery outcome for functional hemispherectomy (ILAE) is 58%; (10 (53%) seizure-free without aura and 1 (5%) 1–3 seizures per year).

## 6. Epilepsy Surgery Outcome according to Histopathology

### 6.1. Mesial Temporal Sclerosis

**Figure 5:**
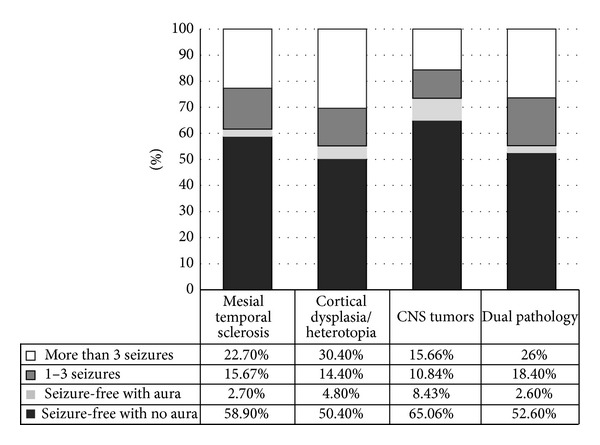
The first-year epilepsy surgery outcome according to histopathology.

**Figure 6:**
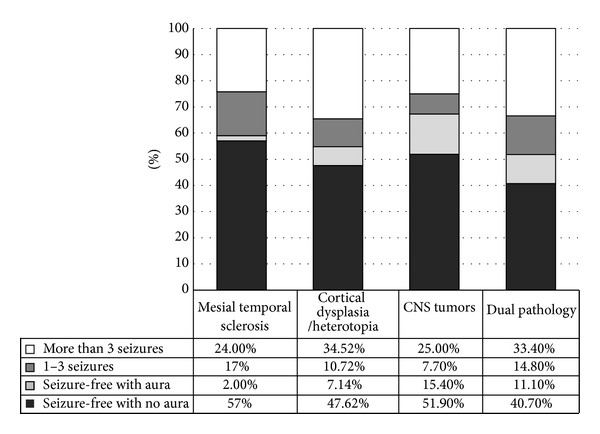
The third-year epilepsy surgery outcome according to histopathology.

According to the ILAE, the favorable outcome for patients with a histopathology diagnosis of mesial temporal sclerosis in the first year following epilepsy surgery is 77.27%. In our cases, 109 patients (58.9%) were seizure-free without auras, 5 (2.7%) were seizure-free with auras, and 29 (15.6%) had 1–3 seizures per year. The favorable outcome for patients in the third year following epilepsy surgery with a histopathology diagnosis of mesial temporal sclerosis, according to the ILAE, is 76%. In our cases, we found 68 patients (57%) were seizure-free without auras, 3 (2.0%) were seizure-free with auras, and 20 (17%) had 1–3 seizures per year. The favorable outcome for patients with a histopathology diagnosis of mesial temporal sclerosis in the fifth year following epilepsy surgery, according to ILAE criteria, is 58%. Our series found 21 patients (42%) were seizure-free without auras, 1 (2%) was seizure-free with auras, and 7 (24%) had 1–3 seizures per year (Figures 5 and 6).

### 6.2. Cortical Dysplasia/Heterotopias

The favorable outcome for patients with a histopathology diagnosis of cortical dysplasia/heterotopias in the first year following epilepsy surgery, according to the ILAE, is 69.6%. Our cases showed 63 patients (50.4%) were seizure-free without auras, 6 (4.8%) were seizure-free with auras, and 18 (14.4%) had 1–3 seizures per year. According to ILAE criteria, the favorable outcome for patients in the third year following epilepsy surgery is 65.48%. In our series, 40 patients (47.6%) were seizure-free without auras, 6 (7.14%) were seizure-free with auras, and 9 (10.7%) suffered from 1–3 seizures per year. According to the ILAE criteria, the favorable outcome for patients in the fifth year following epilepsy surgery is 61.2%. Our study showed 23 patients (47%) were seizure-free without auras, 5 (10.2%) were seizure-free with auras, and 2 (4%) had 1–3 seizures per year (Figures 5 and 6).

### 6.3. Benign CNS Tumors

According to the ILAE, the favorable outcome for patients with a histopathology diagnosis of benign CNS tumors in the first year following epilepsy surgery is 84.3%. Our studies showed 54 patients (65.1%) were seizure-free without auras, 7 (8.43%) were seizure-free with auras, and 9 (10.9%) had 1–3 seizures per year. The favorable outcome for patients in the third year following epilepsy surgery, according to the ILAE, is 75%. In our series, we found 27 patients (51.9%) were seizure-free without auras, 8 (15.4%) were seizure-free with auras, and 4 (7.7%) suffered from 1–3 seizures per year. According to the ILAE, the favorable outcome for patients in the fifth year following epilepsy surgery is 66.5%. Our research found 12 patients (44.5%) were seizure-free without auras, 3 (11%) were seizure-free with auras, and 3 (11%) had 1–3 seizures per year (Figures 5 and 6).

### 6.4. Dual Pathology

The favorable outcome for patients with a histopathology diagnosis of dual pathology in the first year following epilepsy surgery, according to the ILAE, is 73.6%. Our cases showed 20 patients (52.6%) were seizure-free without auras, 1 (2.6%) was seizure-free with auras, and 7 (18.4%) had 1–3 seizures per year. According to the ILAE, the favorable outcome for patients in the third year following epilepsy surgery is 66.6%. In our study, we found 11 patients (40.7%) were seizure-free without auras, 3 (11.1%) were seizure-free with auras, and 4 (14.8%) suffered from 1–3 seizures per year (Figures 5 and 6).

## 7. Epilepsy Surgery Outcomes in Patients with Normal MRI Scans

Thirty-four epilepsy patients with normal MRI brain scans were surgically treated. Surgical outcomes were assessed in 34 out of 35 patients that were seen one year after surgery. The PET scan result of 24 patients showed hypometabolism. The surgical procedures were 17 temporal lobe surgeries, 12 frontal lobe surgeries, 2 parietal and occipital surgeries, 2 corpus callosotomies, and 1 frontal multiple subpial transaction. The epileptogenic region was identified using the clinical semiology and the invasive intracranial EEG recording. The 12- and 36-month epilepsy surgery outcome of 17 temporal lobe surgeries were (53%) and (47%) seizure-free, respectively. The first- and third-year epilepsy surgery outcomes of 15 extratemporal epilepsy surgeries were (47%) and (33%) seizure-free. The histopathology results were 12 focal cortical dysplasia and heterotopias, 12 sclerosis and gliosis, 2 nonspecific, 5 had normal histopathology results and no histopathology tissues were obtained for 2 corpus callosotomies and 1 frontal multiple subpial transaction.

## 8. Discussion 

This study of the Saudi epilepsy surgery series demonstrated that the epilepsy surgery outcomes in our epilepsy center compare well to those from other countries [16]. In this study, we have reported the outcomes of adults and pediatric epilepsy procedures at 12, 36, and 60 months for temporal lobe surgeries and outcomes at 12 and 36 months for frontal, parietal, occipital, multilobar surgery, and hemispherectomy.

The study included the patients that underwent resective surgery in the epilepsy program of Riyadh's KFSHRC between 1998 and 2012. The postoperative outcome was assessed according to a classification adapted from the ILAE. Seizure-free patients without auras, seizure-free patients with postoperative auras, and patients with fewer than three seizures per year were considered to be favorable outcomes based on the recommendations of the ILAE commission report [17, 18].

The literature showed a significant improvement in seizures with temporal and extratemporal epilepsy surgery in children and adults [19–24]. Significant improvements were established based on short- and long-term followups [25, 26]. The estimates of the likelihood of seizure-freedom after epilepsy surgery are 65% to 80% of lesional temporal lobe epilepsy patients [13]. In our series, the favorable outcomes following surgery for temporal lobe epilepsy patients are 79.6%, 74.2%, and 67% for 12, 36, and 60 months, respectively. These results are similar to the reported long-term followup results for surgery for temporal lobe epilepsy [27].

Various studies have examined the rates and predictors of seizure-freedom after resection for frontal lobe epilepsy. There is significant variability in their results due to patient diversity. Across 1,199 patients in 21 studies, the overall rate of postoperative seizure-freedom for at least 48 months (Engel Class I outcome) was 45.1% [28, 29]. In our series, the ILAE Class I–3 outcomes for frontal lobe epilepsy are 62% and 52% for 12 and 36 months, respectively.

Surgery on the parietal and occipital lobes depends largely on the underlying pathology. Previous studies suggest 20% of nonlesional and 75% of lesional parietal lobe cases may be rendered seizure-free by resective surgery [30]. One of the reports in the literature with respect to the outcomes of occipital lobe epilepsy surgery indicated that 46% became seizure-free and 21% had a significant reduction in seizure frequency [31]. Our experience in occipital and parietal lobe epilepsy surgeries indicated that the ILAE class 1–3 surgical outcomes for both first- and third-year followups are 67%.

A total of 53 functional hemispherectomies were performed in our series. Of the 53 procedures, 11 cases were *Rasmussen encephalitis, 25 were hemimegalencephaly and cerebral cortical dysgenesis, and 15 were encephalomalacia. Forty-one of the 53 patients were children.* ILAE class 1–3 surgical outcomes for the first- and third-year followup are 64% and 63%, respectively. The atypical variable in our series is the high ratio (47%) of the *hemimegalencephaly* and cortical *dysgenesis*.

*Hemimegalencephaly* was first described by Sims in 1835 [32]. 15 cases with *hemimegalencephaly that underwent* a functional hemispherectomy were described. Engel class (1a–c) was achieved in 10 out of 15 cases [33]. Another report evaluated 58 children that underwent anatomical, functional, or modified anatomical hemispherectomy for intractable seizures from 1986 to 1995 for seizure control. Seizure control with more than one year of followup revealed a better reduction in seizure frequency in 44 out of 50 (88%). Interestingly, there was no seizure-freedom achievement in this series [34]. Moreover, 23 patients *with Rasmussen encephalitis* underwent surgery [35]. The mean followup was 63.3 months. Eleven patients had total seizure control while 12 individuals persisted with seizures [35].

We did analyze the surgical outcome based on the histopathology diagnosis. CNS and MTS patients had a better outcome than those with cortical dysplasia (CD) or dual pathology at 1 and 3 years after surgery. In the Irish epilepsy surgery experience, MTS patients had a significantly better outcome than those with CD, a CNS tumor, and other pathology groups at 1, 2, and 5 years after surgery [36]. Dual pathology is recognized in the histopathology of epileptic tissues [37, 38]. Our results demonstrated that 7.5% of the total histopathology results had two or more different potentially epileptiform abnormalities. It is remarkable that the least seizure-free outcome after 3 years did occur in the cases with dual pathology.

In our series, 34 cases of refractory nonlesional focal epilepsy cases have been operated upon with encouraging results, although the number is too small to allow indisputable analysis; however, our results match with some of the reported similar literature. Jayakar et al. studied 102 patients with nonlesional intractable partial epilepsy that underwent excisional surgery. At the 2-year followup, 44 out of 101 patients were seizure-free, and 15 experienced greater than 90% reduction [39].

In conclusion, the epilepsy surgery outcome in our comprehensive epilepsy program is comparable to the international standard. In our series, the most favorable outcomes are achieved with temporal epilepsy surgery procedure, histopathological diagnosis of mesial temporal sclerosis, benign CNS tumor, and cortical dysplasia, while the least favorable outcomes occurred with multilobar surgery procedure, dual pathology, and normal MRI brain patients.

## Figures and Tables

**Table 1:** ILAE classification of surgical outcome with respect to epileptic seizures.

Outcome classification	Definition
1	Completely seizure-free; no auras
2	Only auras; no other seizures
3	One to three seizure days per year; ±auras
4	Four seizure days per year to 50% reduction of baseline seizure days; ±auras
5	Less than 50% reduction of baseline seizure days to 100% increase of baseline seizure days; ±auras
6	More than 100% increase of baseline seizure days; ±auras
